# First Report of Complete Genome Analysis of Multiple Drug Resistance *Proteus mirabilis* KUST-1312 Isolate From Migratory Birds in China: A Public Health Threat

**DOI:** 10.1155/2024/8102506

**Published:** 2024-10-03

**Authors:** Jiayu Gao, Shufa Liu, Sadia Bano, Xueshan Xia, Zulqarnain Baloch

**Affiliations:** Faculty of Life Science and Technology, Yunnan Provincial Center for Molecular Medicine, Kunming University of Science and Technology, Kunming 650500, China

**Keywords:** antibiotics-resistant genes, chromosome, migratory birds, *Proteus mirabilis*

## Abstract

*Proteus mirabilis*, a gram-negative bacterium, poses a significant public health threat due to its multidrug-resistant (MDR) characteristics. Here, for the first time, we report the isolation and comprehensive genome analysis of an MDR strain, *P. mirabilis* KUST-1312, obtained from migratory birds in Yunnan Province, China. A total of 65 samples, including migratory bird feces, soil, and water from Dianchi Lake, were collected. Standard microbiological techniques were employed to isolate the *P. mirabilis* KUST-1312 strain from these samples. Genomic sequencing was conducted using a hybrid assembly strategy, combining Illumina and Oxford nanopore sequencing technologies. Phenotypic testing revealed the MDR nature of *P. mirabilis* KUST-1312, displaying resistance to various antibiotics except gentamicin and Cefotaxime. Notably, 15 antimicrobial resistance genes (ARGs), including *aph*(*3′*)-*Ia*, *cat*, *tet*(*J*), *bleO*, *dfrA12*, *aadA2*, *AAC*(*3*)-*IId*, *bla-TEM-1B*, *erm*(*42*),*aph*(*6*)-*Id*, *blaPER-1*, *sul2*, *aph*(*3′'*)-*Ib*(2copies), and *aph*(*3′*)-*VIb*, were identified on a single chromosome. These 15 ARGs were dispersed along three MDR regions, and the boundaries of these regions were consistently flanked by copies of insertion sequences and also contained other genetic elements. Phylogenetic analysis revealed the close relation of *P. mirabilis* KUST-1312 with environmental and clinical isolates reported from other continents rather than with Asian isolates. In conclusion, this study reports the first-ever isolation of an MDR *P. mirabilis* KUST-1312 strain from migratory birds globally, particularly in China. There is a need to explore further its prevalence in detail in various ecological niches, including migratory birds.

## 1. Background

*Proteus mirabilis*, a member of the *Morganellaceae* family within the order *Enterobacterales*, is a gram-negative, rod-shaped, facultative anaerobe known for its diverse metabolic activities. While capable of fermenting maltose, it cannot ferment lactose, establishing its distinct biochemical profile. Present in the human gastrointestinal tract, *P. mirabilis* is an opportunistic pathogen with the potential to cause various infections, including gastroenteritis, keratitis, wound infections, and urinary tract infections [1–4]. This bacterium is widespread among diverse hosts, including humans, domestic and wild animals, and their environments, contributing to the global dissemination of antibiotic resistance [5, 6]. The emergence of multidrug-resistant (MDR) *P. mirabilis* poses a significant challenge in clinical settings worldwide [7–14], exhibiting resistance to antibiotic classes such as nitrofurans, Colistin (CL), tetracycline, tigecycline [12], and *β*-lactams [15]. Notably, it has been identified as a causative agent in a significant percentage of urinary tract infections, particularly in complicated cases, with higher prevalence and mortality rates in specific patient groups [16–19].

*P. mirabilis* is a known pathogen with a well-documented impact on human and animal health and has never been previously identified in migratory birds (especially the MDR strain) worldwide. Despite their crucial role in global ecosystems, migratory birds have not been a primary focus in studies examining the transmission and spread of uncommon MDR pathogens like *P. mirabilis*. This lack of research has led to a substantial gap in understanding how migratory birds may act as vectors for these resistant strains, contributing to their dispersion across regions and potentially posing public health risks. Filling this knowledge gap is essential, as identifying and characterizing MDR *P. mirabilis* in migratory birds can provide insights into the spread of antibiotic resistance and help in devising strategies to mitigate its impact on both wildlife and human health. Here, for the first time, we report the isolation and comprehensive genome analysis of an MDR strain, *P. mirabilis* KUST-1312, obtained from migratory birds in Yunnan Province, China.

## 2. Method

### 2.1. Sample Collection and Isolation

Dianchi Lake (102°36′‒102°47′E, 24°40′‒25°20′N) is an ancient tectonic lake on the Yunnan-Guizhou Plateau within the Yangtze River Basin. Covering 308.6 km^2^, it is China's 6th-largest freshwater lake and the largest on the plateau. The lake is split into two sections by a semi-artificial dam: the northern section, Caohai, spans 10.7 km^2^ with a mean water depth of 2.5 m, while the southern section, Waihai, covers 297.9 km^2^ with a mean depth of 4.3 m. Because of its size and ecological significance, Dianchi Lake is an important habitat for many bird species, particularly migratory birds, providing a rich environment for studying their behavior and examining the potential spread of pathogens. Therefore, a total of 65 samples, comprising migratory bird feces (*n* = 37), soil (*n* = 11), and water (*n* = 17), were collected from Dianchi Lake in Kunming and transported at 4°C to the laboratory. Fecal samples were aseptically transferred to glass tubes with PBS and streaked on nutrient agar plates, while other samples were directly streaked. After a 24-h incubation at 37°C, species identification utilized standard biochemical tests and the API20E system.

### 2.2. Antimicrobial Susceptibility Testing

Antimicrobial susceptibility testing was conducted using the broth microdilution method, by following Clinical and Laboratory Standards Institute (CLSI) protocols. Twenty-Seventh Informational Supplement M100-S27. Wayne; CLSI [20]. “Performance Standards for Antimicrobial Susceptibility Testing) with *E. coli* ATCC 25922 as the quality-control strain. A range of antibiotics was tested: Amoxicillin (AMX), Tulathromycin (TUL), Ciprofloxacin (CIP), Enrofloxacin (ENR), Kanamycin (KAN), Florfenicol (FLOR), CL, Oxytetracycline (OXY), Tiamulin (TI), Sulfadiazine (SUL), Doxycycline (DOX), Ampicillin (AMP), Cefotaxime (CTX), and Gentamicin (GM). The results were interpreted according to CLSI criteria where needed (Clinical and Laboratory Standards Institute. Performance Standards for Antimicrobial Susceptibility Testing. Twenty-Seventh Informational Supplement M100-S27. Wayne; CLSI [21].

### 2.3. DNA Extraction

DNA extraction was conducted using the Bacterial Genomic DNA kit from Beijing Zoman Biotechnology Co., Ltd, following the manufacturer's specified protocol. After extraction, DNA concentration was quantified with a Qubit v2.0 Fluorometer (Thermo Fisher Scientific, USA) to ensure accuracy. To confirm the integrity of the extracted DNA, we ran it on an agarose gel, checking for quality and ensuring there were no signs of degradation.

### 2.4. PCR Amplification of Antibiotic-Resistant Genes

Bacterial strain identification was carried out using 16S rRNA primers (27F and 1492R), along with primers specific to *P. mirabilis*. Further, we targeted following ESBL genes (bla_*TEM*_, bla_*SHV*_, bla_*CTX-M*_, bla_*VEB*_, and bla_*PER*_), fluoroquinolone resistance genes (*qnr*A and *qnr*B), tetracycline resistance genes (tet(A), *tet*(B)), and carbapenemase genes for imipenem resistance (bla_*IMP*_ and bla_*NDM−1*_) for PCR amplification. PCR was carried out with a 50 *µ*L reaction mixture, which contained 6 *µ*L of the DNA extract, 1 *µ*L each of 10 *µ*M forward and reverse primers, 25 *µ*L of the master mix, and 15 *µ*L of distilled water (ddH_2_O). The PCR-amplified products were analyzed on a 1%–2% agarose gel to ensure proper amplification and then purified using a Tiangel purification kit (Tiangen, Beijing, China). The purified DNA was subsequently sequenced with the ABI PRISM Big Dye Terminator Cycle Sequencing Ready Reaction kit (Invitrogen, Beijing, China) and processed on an ABI 310 DNA analyzer. The resulting nucleotide sequences were aligned and compared using GenBank's BLAST 2.0 server to confirm the identity and match with known sequences.

### 2.5. Long- and Short-Read Sequencing and Analysis

To investigate the genomic characteristics of *P. mirabilis* KUST1312, the complete genome sequence of strain was obtained with a hybrid assembly strategy combining short-read Illumina sequencing (Illumina, 2 × 150 bp paired-end reads) and long-read Oxford nanopore sequencing technologies using Unicycler v0.4.8, and Flye v2.7.1. [22, 23]. Both generated sequences were analyzed for antimicrobial resistance genes (ARGs), insertion sequences (ISs), plasmid, and CRISPR loci using AMRFinder v3.8.4., ISfinder, PlasmidFinder, and CRISPRfinder [24–27]. Sequence comparison of chromosomes was conducted using Mauve as previously described [28, 29].

### 2.6. Phylogenetic Analysis

The genomic sequences of representative Proteus isolates worldwide were obtained from GeneBank. Using Snippy v4.6.0 (https://github.com/tseemann/snippy), assemblies from all isolates were mapped to the reference sequence ATCC 51959 (accession number GCA_016844085.1). Single nucleotide polymorphisms were identified, and recombinant regions were excluded using Gubbins v2.4.1. RAxML v8.2.12 (GTRGAMMA substitution model) with 100 bootstrap replicates was employed to construct a phylogenetic tree. The resulting tree was visualized using iTOL and Chiplot.

## 3. Results

In this study, only a single *P. mirabilis* KUST-1312 strain was isolated from all 37 migratory bird feces samples, and none of the soil or water samples were detected to be positive for *P. mirabilis*. The phenotypic susceptibility testing indicated that *P. mirabilis* KUST-1312 is resistant to a range of antibiotics, with only GM and CTX showing susceptive. The resistance results are as follows: AMX (512), AMP (512 *µ*g/mL), KAN (128 *µ*g/mL), TUL (512 *µ*g/mL), CL (512 *µ*g/mL), SUL (512 *µ*g/mL), ENR (16 *µ*g/mL), CIP (64 *µ*g/mL), TI (512 *µ*g/mL), OXY (256 *µ*g/mL), DOX (64 *µ*g/mL), and FLOR (64 *µ*g/mL). After phenotypic characterization, various genes, including bla-TEM-1B, *sul2*, *and blaPER-1*, were detected with PCR in the *P. mirabilis* KUST-1312 strain.

**Figure 1:**
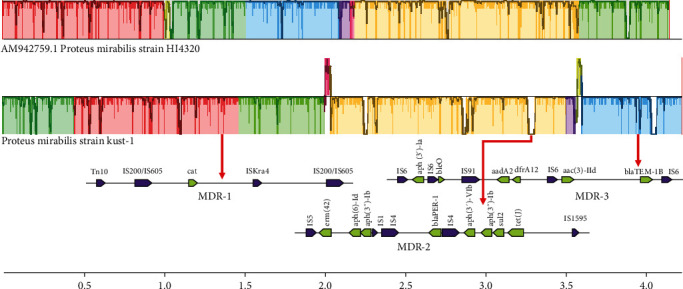
Comparisons of chromosomal synteny and content were conducted among nine fully sequenced *P. mirabilis* KUST-1312 isolates. The arrows depicted in the figure indicate the direction of gene transcription. Genes are color-coded based on their sequence and function. Please note that this figure is presented in color in the online version and in black and white in the print version.

The genomic analysis identified a single chromosome (4,151,496 bp, 39.01% GC content) and an unclassified plasmid (36,007 bp) with no resistance genes. Fifteen ARGs were found on a single chromosome and categorized into three MDR regions (Figure 1). The first region exclusively contained the chloramphenicol resistance gene (*cat*). The 2nd MDR region contains antibiotic resistance genes, including *aph*(*3′*)-*Ia*, *bleO*, *dfrA12*, *aadA2*, *AAC*(*3*)-*IId*, and *bla-TEM-1B*. The 3rd MDR region harbored seven antibiotic genes, including *tet*(*J*), *erm*(*42*), *aph*(*6*)-*Id*, *blaPER-1*, *sul2*, *aph*(*3′'*)-*Ib* (two copies), and *aph*(*3′*)-*VIb*). The 2nd and 3rd MDR region's boundaries were commonly flanked by copies of ISs. Furthermore, the MDR regions consisted of multiple ARGs interspersed with different ISs. MDR-1 and MDR-2 2 contain transposons (tn10 and two copies of tn3). Additionally, a complete integron (integrase intI and attC site) of 2915 bp was detected by IntegronFinder.

**Figure 2:**
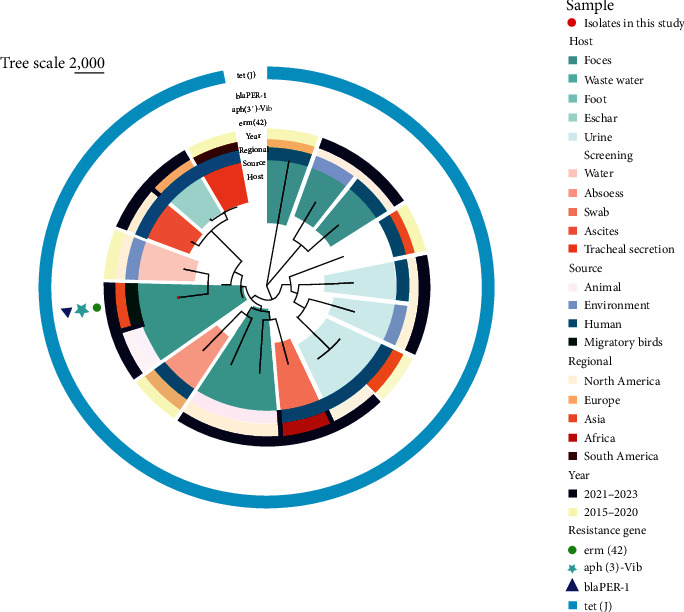
The phylogenetic structures of *P. mirabilis* KUST-1312 in a global context are illustrated in the maximum likelihood tree. The tree delineates the phylogenetic relationships among 18 *P. mirabilis* isolates unique to this study (highlighted by the red points) along with 17 global *P. mirabilis* isolates. Internal nodes on the tree are labeled with circles, indicating branches with greater than 70% bootstrap support. Metadata are visually represented in concentric circles as follows: (1) Host, (2) Source, (3) Regional, (4) Year, and (5–8) Presence of ARGs (*emr*(*42*), *aph*(*3′*)-*Vib*, bla_*PER−1*_, and *tet*(*J*)). It's important to note that this figure is presented in color in the online version and in black and white in the print version.

For the phylogenetic analysis, we downloaded 17 existing genome sequences from various continents, selecting them based on sample type, source, and year of isolation (see File S1). Our analysis revealed that the *P. mirabilis* KUST-1312 isolates from the current study is closely associated with environmental and clinical isolates reported from other continents rather than with Asian isolates. Bayesian hierarchical clustering of the core genome alignment demonstrated distinct lineages, as illustrated in Figure 2.

## 4. Discussion

*P. mirabilis* is frequently reported as a causative agent in urinary tract infections [13] and food-borne illnesses in humans. Additionally, it has also been reported in farm animals [4]. Moreover, in 2021, 25 MDR *P. mirabilis* isolates were isolated from different wild animals in China [30]. Here, for the first time, we report the isolation of MDR *P. mirabilis* KUST-1312 from migratory birds in Yunnan Province, China. This strain exhibited resistance to 12 tested antibiotics, with the exceptions of GM and CTX. Generally, *P. mirabilis* is naturally resistant to polymyxin, tigecycline, and tetracycline [30]. However, it is sensitive to other antibiotics, including aminoglycosides, macrolides, chloramphenicol, carbapenems, etc. *P. mirabilis* KUST-1312 isolate showed a resistant phenotype to various classes, which is in line with previous studies [30].

Further, we reported the complete genome sequence of *P. mirabilis* KUST-1312, obtained from the feces of a migratory bird. The genomic analysis identified a single chromosome (4,151,496 bp, 39.01% GC content) [31] and an unclassified plasmid (36,007 bp) with no resistance genes in *P. mirabilis* KUST-1312-plasmid, sharing 99.82% similarity with a plasmid previously reported from China (“genomic characterization of multidrug-resistant Proteus isolates”). We identified the integration of multiple ARGs into the chromosome, facilitated by mobile elements such as ISs and transposons, which is in line with previous studies in China [13, 30]. When ARGs are integrated into the chromosome, the stability of these resistance traits is generally higher compared to when they are located on plasmids. Chromosomal ARGs are less likely to be lost through the process of plasmid segregation during cell division. This stability can mean that resistance is more reliably passed down through generations of bacteria, making these strains more robust in maintaining their resistance profile. The IS elements, transposons, and other MGEs have a significant role in the spread of antibiotic resistance. They can carry ARGs to new locations within a genome, leading to the emergence of novel resistance profiles. Additionally, by transferring ARGs across plasmids and chromosomes, they facilitate the exchange of resistance genes among different bacterial populations, contributing to the rise of MDR and extensively drug-resistant bacteria.

We found the presence of six aminoglycosides resistant genes *aadA1*, *aph*(*6*)-*Id*, *aph*(*3′'*)-*Ib* (two copies), *aph*(*3′*)-*VIb*, *AAC*(*3*)-*IId*, and *aph*(*3′*)-*Ia*, 2 *β*-lactams resistant genes, such as bla*TEM-1*, and *blaPER-1*, 1 sulfonamide-resistant gene *sul2*, 1 Trimethoprim gene *frA12*, 1 tetracycline-resistant gene. We found the presence of aminoglycosides-resistant genes gene high in a single isolate compared to previously reported studies [4, 13, 30, 32]. Further, the majority of aminoglycoside-resistant genes have already been reported in *P. mirabilis* isolates in China. However, *aph*(*3′*)-*VIb* has never been reported in *P. mirabilis* before in China. There is a need to explore the possible transmission source of *aph*(*3′*)-*VIb*. The ESBL-resistant genes, such as TEM, were first reported in 1983 [32], and *P. mirabilis* is highly represented as an ESBL host. PER *β*-lactamases are a group of ESBL enzymes. To date, seven variants of the *blaPER* gene have been identified [30]. Of these, three variants—*blaPER-3*, *blaPER-4*, and *blaPER-5*—differ from *blaPER-1* by only one amino acid. The *blaPER-7* variant has four amino acid differences compared to *blaPER-1*. On the other hand, blaPER-2 shows a 14% difference in its amino acid sequence compared to *blaPER-1*, while *blaPER-6* is also 8% different compared to *blaPER-2*. These differences suggest a range of variability within the PER *β*-lactamase family, indicating potential variations in enzyme function and antibiotic resistance profiles. *blaPER-1* and *blaPER-7* have been reported from Europe and Asia [33–38], *blaPER-2* has been reported in the South American, Asian, African, and European continents [33, 39–41], and *blaPER-6* has only been reported in Paris Europe [42]. *blaPER-1* has been reported in China; however, very limited studies have reported the prevalence of blaPER-3 and blaPER-4 in China [43]. However, no data are available about the epidemiology of *blaPER-5* variants [38]. However, our study reveals a unique case (Migratory bird); limited studies reported *blaPER* genes in clinical isolates from China [43], and a single isolate from a Bighead Carp sample in Shanghai has been reported positive for these resistance genes (“Microbe sample from *Proteus mirabilis*, 1035,”). Given that migratory birds transcend borders, they serve as potential vectors for the dissemination of antibiotic-resistant microbes and elements across geographical boundaries. Consequently, this study underscores the imperative of conducting surveillance to monitor fluctuations in the prevalence and distribution of ARGs and other resistance elements in migratory birds and their corresponding environments.

There are several limitations to the current study. First, the sample collection was limited to migratory birds, along with associated soil and water sources. This exclusion of samples from humans, local animals, and resident bird species restricts the study's ability to fully understand the transmission dynamics and potential pathways of antibiotic-resistant bacteria across different ecosystems. This focused approach may lead to an incomplete representation of the broader context in which AMR develops and spreads. The second limitation is that the study did not assess the expression levels of the identified ARGs. While the presence of these genes indicates potential resistance, actual expression in various conditions would provide deeper insights into their functional roles and clinical relevance. Without this information, it is difficult to determine how active these resistance mechanisms are in different environments or under varying selective pressures, leaving gaps in understanding the real-world impact of these genes on bacterial resistance.

In conclusion, we have successfully isolated a critically important MDR *P. mirabilis* strain from migratory birds for the first time in the world. This strain, identified as *P. mirabilis* KUST-1312, contains 15 ARGs spread across MDR regions on a single chromosome. The mechanism of resistance is elucidated by the integration of these ARGs into the chromosome through mobile genetic elements. Given the role of migratory birds as potential reservoirs for ARGs, it is crucial to maintain ongoing monitoring of *P. mirabilis* within these bird populations and their related ecosystems. Our findings underscore the global implications of antibiotic resistance and call for coordinated international efforts to understand and tackle this pressing public health challenge.

## Data Availability

The complete nucleotide sequences of the complete genome of *P. mirabilis* isolates reported in this study were submitted to GenBank and shared one CP141835.

## Supporting Information

Supporting Information File S1: Seventeen *P. mirabilis* isolates SRA files belonging to various countries were submitted from different countries and used for phylogenetic analysis.
