# Supplementary material for: First Report of Complete Genome Analysis of Multiple Drug Resistance Proteus mirabilis KUST-1312 Isolate From Migratory Birds in China: A Public Health Threat
Source: Transbound Emerg Dis. 2024 Oct 3;2024:8102506. doi: 10.1155/2024/8102506 (PMC12016733; doi:10.1155/2024/8102506)
Supplement: Supporting Information — File S1: Seventeen P. mirabilis isolates SRA files belonging to various countries were submitted from different countries and used for phylogenetic analysis. [file 8102506.f1.pdf]

tree ID	#Organism group	Strain	Isolate identifiers	Serovar	Isolate	Create date	Location	Isolation source	Isolation type	SNP cluster	Min-same	Min-diff	BioSample
SRR25798613	Providencia alcalifaciens	G2304450_a	"G2304450_a","SRS18744012"		PDT001869108.1	2023-08-30T20:42:34Z	USA:WV	canine	environmental/other				SAMN37198773
SRR24510086	Providencia alcalifaciens	GT23-0293	"GT23-0293","SRS17620729"		PDT001729243.1	2023-05-11T20:56:55Z	USA:SC	wastewater	environmental/other				SAMN35047792
SRR24313739	Providencia alcalifaciens	V1	"SRS17439553","V1"		PDT001792668.1	2023-06-28T03:03:23Z	Canada: Guelph	wastewater	environmental/other	PDS000150813.1	1		SAMN34382669
SRR20047401	Providencia alcalifaciens	2022GN-00288	"2022GN-00288","SRS13751771"		PDT001355509.2	2023-06-07T10:27:46Z	USA	Foot	clinical				SAMN29599262
SRR18714278	Providencia alcalifaciens		"HURS-173346","SRS12571266"		PDT001529526.1	2022-12-16T16:51:50Z	Spain	Eschar	clinical	PDS000109057.2	1		SAMN27518690
SRR13926740	Providencia alcalifaciens	800.362.2	"800.362.2","SRS8427564"		PDT000984087.1	2021-03-12T21:29:40Z	USA: CA	feces (Canis lupus familiaris)	environmental/other				SAMN18243132
SRR12517477	Providencia alcalifaciens	hkcpe42	"SRS7259686","hkcpe42"		PDT000881395.1	2020-11-06T19:24:48Z	Hong Kong	urine	clinical	PDS000075324.3	43	22	SAMN15904711
SRR11531469	Providencia alcalifaciens	microbial	"RZ142","SRS6468430","microbial","rectal swab"		PDT000751239.1	2020-05-30T16:15:06Z	Kuwait	SCREENING	clinical				SAMN14589629
SRR11103430	Providencia alcalifaciens	D6	"D6","SRS6160985"		PDT001529605.1	2022-12-16T17:39:58Z		water	environmental/other	PDS000127956.1	7		SAMN14128547
SRR7822181	Providencia alcalifaciens		"PmOXA23-18","SRS3767927"		PDT001529716.1	2022-12-16T17:40:16Z	France: Besancon	Abscess	clinical	PDS000049706.4	2		SAMN10038500
SRR6037657	Providencia alcalifaciens	CCUG 70746	"A4_Pmi","CCUG 70746","CCUG:70746","SRS2514200"		PDT000326683.1	2018-06-06T14:07:33Z	Sweden: Gothenburg	feces	clinical	PDS000049647.1	0		SAMN07602702
ERR10432835	Providencia alcalifaciens		"DTU_2021_1002597_1_SI_ZMB_LUS_UTH_057","ERS13618755"		PDT001493790.1	2022-11-22T19:46:20Z	Zambia	swab (wound)	clinical	PDS000124883.1	2		SAMEA111506393
SRR25910964	Providencia alcalifaciens	2023EP-00201	"2023EP-00201","SRS18801696"		PDT001873071.1	2023-09-05T16:57:00Z	USA	catheter tip	clinical				SAMN37282413
SRR16292909	Providencia alcalifaciens		"2021JQ-00171","SRS10528768","whole organism"		PDT001148881.2	2023-05-19T22:56:02Z	USA	Fluid, Ascites	clinical	PDS000097166.5	35		SAMN22216698
SRR25438672	Providencia alcalifaciens		"BWH_195_P_mir","SRS18433678"		PDT001830972.1	2023-07-27T18:48:20Z	USA:Boston	urine	clinical				SAMN36729017
SRR25730270	Providencia alcalifaciens	2023EL-00829	"2023EL-00829","SRS18690298"		PDT001868580.1	2023-08-29T03:18:34Z	USA	urine	clinical	PDS000091362.10	0		SAMN37112976
QGGA01.1	Providencia alcalifaciens	LBUEL-H11	"LBUEL-H11","Proteus mirabilis LBUEL-H11"		PDT000321863.1	2018-05-26T02:20:55Z	Brazil:Londrina	tracheal secretion	clinical				SAMN09222159

Supplementary file 1: 17 Proteus mirabilis isolates SRA files were downloaded belong to various countries were submitted from different countries and used for phylogenetic analysis
